# Pelvic squamous cell carcinoma of unknown primary origin with hydronephrosis and ureteral stricture: A case report

**DOI:** 10.1097/MD.0000000000037057

**Published:** 2024-01-26

**Authors:** Wenjun Meng, Yuchen Gao, Lu Pan, Guowei Zhao, Qi Chen, Lian Bai, Rujun Zheng

**Affiliations:** a Department of Biotherapy, Cancer Center, West China Hospital, Sichuan University, Chengdu, China; b Department of General Surgery, Beijing Mentougou District Hospital, Beijing, China; c Department of Gastrointestinal Surgery, Yongchuan Hospital, Chongqing Medical University, Chongqing, China.

**Keywords:** cancer of unknown primary, case report, pelvic tumor, poorly differentiated squamous cell carcinoma

## Abstract

**Background:**

Cancer of unknown primary (CUP) is a very challenging disease, accounting for 2% to 9% of all new cancer cases. This type of tumor is a heterogeneous tumor whose primary site cannot be determined by standard examination. It has the characteristics of early metastasis, strong aggressiveness, and unpredictable mode of metastasis. Studies have shown that there is no consensus on the treatment of CUP and that there is a wide range of individual differences. In most cases, surgical removal of tumor is the most typical treatment for pelvic tumors. Herein, we report a case of a large pelvic tumor of unknown origin that had compressed the sigmoid colon and ureter and was completely removed by surgery. Postoperative diagnosis was pelvic metastatic squamous cell carcinoma.

**Case summary:**

A 68-year-old man with pelvic tumor who initially complained of recurrent low back pain and painful urination. The mass was initially diagnosed as a pelvic tumor of unknown origin. The patient underwent complete resection of the tumor by laparotomy. The tumor was pathologically diagnosed as squamous cell carcinoma.

**Conclusion:**

Based on the treatment experience of this case, surgery alone cannot improve the poor prognosis of CUP. Since chemotherapy and immunotherapy have achieved promising efficacy in various cancers, and immunotherapy has the characteristics of low side effects and good tolerability, we recommend that patients with CUP should receive chemotherapy and/or immunotherapy for better survival outcomes.

## 1. Introduction

Cancer of unknown primary (CUP) is defined as the metastatic tumor of unknown primary origin after extensive laboratory and clinical investigations.^[1]^ Since CUP has developed into a metastatic tumor at the initial diagnosis, it is usually highly malignant with poor prognosis.^[2]^ CUP is a heterogeneous malignancy that accounts for 2% to 9% of all newly diagnosed cancer cases.^[3]^ Although the proportion of CUP in all malignancies has fallen to 1% to 2% due to rapid advances in tumor genomic profiling and diagnostic methods, the overall survival (OS) rate of CUP has not improved significantly over the past 10 years.^[4]^ The majority of CUP patients have a poor prognosis, with a median OS of only 6 months.^[5]^ The primary origin of most CUPs cannot be confirmed even after a comprehensive diagnostic work-up. Primary tumor origin can be successfully identified in <30% of patients initially diagnosed with a CUP, while the rate of primary tumor identification is only 50-80% in autopsy series of CUP patients.^[6]^ Despite recent advances in diagnostic techniques and treatment strategies for a variety of cancers, CUP treatment modalities vary in clinical practice due to different signs, symptoms and spread of CUP. In other words, there is currently no consensus on the standard treatment for CUP due to its heterogeneity. Additionally, despite several studies using targeted therapy or immunotherapy in patients with CUP, the survival outcomes of these tailored therapy remain controversial.^[2]^

We recently performed surgical resection of a male patient with multiple pelvic metastases of unknown origin. Computed tomography (CT) showed that the pelvic tumor had invaded the rectum, sigmoid colon, bladder, and prostate. The patient underwent surgical resection of the rectum and sigmoid colon. Pathological examination revealed that the tumor originated from squamous epithelial cells. However, neither gastroscopy nor colonoscopy identified the primary origin of the tumor. In this case study, we reported our experience with the diagnosis and treatment of a patient with CUP. In addition, relevant literature was briefly reviewed.

## 2. Case presentation

### 2.1. History of presentation and investigation

A 68-year-old male patient began to present lower back pain and persistent abdomen flatulence pain about 6 months ago. There was a slight improvement when the patient lay flat. In addition, the patient had symptoms of urinary pain, but he did not receive any treatment. More than 2 months before admission, these symptoms got worse to hematuria, and the symptoms of urinary frequency, urgency, and dysuria tend to occur together. Therefore, he was admitted to our hospital, the Yongchuan Hospital of Chongqing Medical University, for examination and treatment. The patient’s temperature was normal and he had no symptoms of cough or sputum. He did not present with symptoms of intestinal obstruction in which the anus stopped venting and defecating. No significant lower limb swelling was observed on the day of admission. The patient reported that he had a history of hypertension for more than 1 year, a history of peptic ulcers for several years, and had occasional symptoms of stomach pain and bloating. In addition, the patient had a long history of smoking and drinking. No family history presents compelling evidence links to the CUP.

The physical examination of the patient revealed: unsatisfactory palpability in both kidneys, no percussion pain in both kidney areas, no percussion pain in ureteral region, no percussion pain in bladder area. In addition, the foreskin and penis were diagnosed as normal, with no induration and no redness of external urethral meatus. Palpation of the scrotum and bilateral testes revealed normal epididymis. The patient’s laboratory examinations showed a slightly elevated carcinoembryonic antigen level of 4.71 ng/mL (reference: 0–4.5 ng/mL). Moreover, the levels of cancer antigen 19-9, cancer antigen 242, and total prostate specific antigen were within normal range (25.09 U/mL, 13.97 U/mL, and 1.29 ng/mL, respectively).

**Figure 1.**
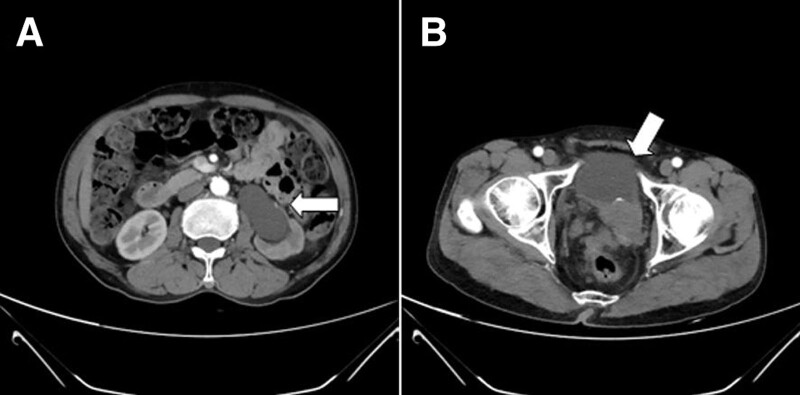
Contrast-enhanced computed tomography (CT) image of the patient with pelvic tumor. (A) Arterial phase scan showing proximal ureter and dilated left hydronephrosis. (B) The white arrow indicates soft tissue mass behind the left bladder. The lesion had invaded the posterior wall of the bladder, and was poorly demarcated from the adjacent bowel.

The CT urography showed a mass soft tissue shadow in the left side of pelvic cavity, suggesting that the malignant tumor had invaded the adjacent rectum, sigmoid colon, bladder, and prostate. Color ultrasound showed that the patient had full dilatation of the left ureter, left hydronephrosis, right kidney stones, and hepatic cysts. A hypoechoic mass was detected in the lower segment walking area, suggesting that the lesion may originate from the ureter. In addition, a hyperechoic mass was detected at the junction with the bladder, suggesting the presence of dense calcification. Moreover, a CT scan revealed a dilated left hydronephrosis and left hydroureter (Fig. 1A) and a soft tissue mass in the left posterior side of the bladder (Fig. 1B), which might be a neoplastic lesion. The lesion wrapped around the left ureter and secondary proximal ureter, invaded the posterior wall of the bladder, and was poorly demarcated from the adjacent intestine. In addition to partial enlargement of multiple lymph nodes in the pelvic, and stones were found in both kidneys. The CT scan also showed calcification in the abdominal aorta and bilateral iliac artery wall, multiple cysts in the liver, and mild dilatation of the intrahepatic bile duct. Gastroscopy and enteroscopy further revealed that the patient had a duodenal bulbar ulcer, chronic non-atrophic antral gastritis, esophagogastric mucosal heterotopia, and multiple polyps in the colorectum.

### 2.2. Diagnosis

Even after detailed examinations by our multidisciplinary team, the nature of the pelvic tumor was unclear, and the origin of the malignancy could not be determined. Therefore, the final diagnosis of the malignancy was a CUP.

### 2.3. Management

Due to extensive invasion, the patient was advised to undergo early therapeutic interventions, including pelvic tumor resection, partial cystectomy, and postoperative chemotherapy and immunotherapy. The patient underwent pelvic tumor resection, retroperitoneal lymph node dissection, partial cystectomy, and sigmoidectomy as planned. The patient did not continue to receive any chemotherapy, immunotherapy or targeted therapy due to economic reasons. Unfortunately, the patient died of metastatic disease 3 months after surgery. For these CUPs, because there is no clear target, only empirical targeted therapy can be done.

### 2.4. Histopathological examination

**Figure 2.**
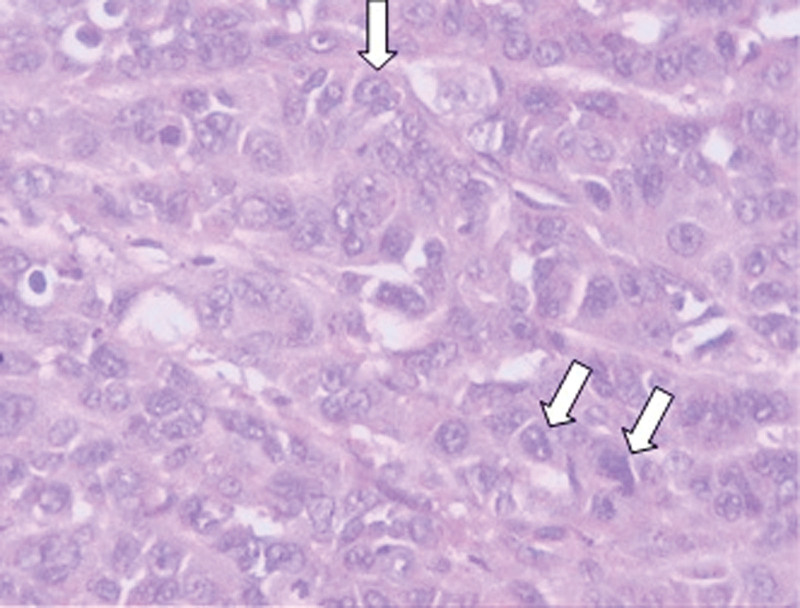
Histopathological staining of the surgically resected tissues. The magnification power is 20 × 10. Hematoxylin and eosin staining showing poorly differentiated pelvic squamous cell carcinoma. The metastatic carcinoma was found in the lymph nodes at the root of the inferior mesenteric vessels.

Postoperative histopathological examination showed that the malignancy was poorly differentiated pelvic carcinoma, which belonged to poorly differentiated squamous cell carcinoma (Fig. 2). Pathological findings did not report bladder squamous cell carcinoma. A small amount of cancer can be seen around the bladder, which is speculated to be caused by pelvic tumor invasion. The final immunohistology chemistry results are CK(+), EMA(+), CK5/6(+), P40(+), P63(+), CK7(−), CK20(−), SATB2(−), CDX2(−), Villin(−), GATA3(−), UP(3), Syn(−), CgA(−), Ki67(+, 60%).

## 3. Discussion

Pelvic neoplasms are abnormal mass in the pelvic cavity. Most of them are located in a woman’s uterus or ovaries, while they can also come from other organs or tissues in the pelvic cavity, such as the abdominal wall, rectum, bladder and the prostate gland. CUP is a heterogeneous metastatic cancer in which the primary tumor cannot be confirmed after a series of standardized examinations. The clinical manifestations of CUP are diverse, characterized by early metastasis, strong invasiveness, and unpredictable modes of metastasis. Due to atypical symptoms and slow growth, most patients have resulted in locally advanced or the tumor has grown to a substantial size at the time of initial detection. When a tumor grows large enough, it presses on certain organs of the body. Giant pelvic tumors are rarely reported. A recent case in Turkey described a 36-year-old female patient who was admitted with abdominal distension and abdominal pain, and CT confirmed that the tumor in the pelvic cavity had become large enough to compress the colon.^[7]^ Our patient had developed symptoms of low back pain and urinalgia 6 months before admission, confirming that the pelvic tumor was slow-growing and asymptomatic. As a result, patients with CUP have poor OS rates, with a median survival of only 6 to 12 month.^[8]^ According to published cancer statistics, although there are up to 30,270 cases diagnosed as CUP in the United States in 2020, there are few reports of CUP in China.^[9]^ As the number of patients with CUP increases, identifying the source of the tumor becomes an essential step.

The current standard examinations mainly include a detailed history, physical examination, complete blood count, comprehensive metabolic and electrolyte testing, urinalysis, fecal occult blood test, and chest radiograph, as well as a complete pathologic assessment of biopsied tissue. For cancers diagnosed at an advanced stage, the location of the primary tumor can be determined by tests that directly diagnose it. However, despite comprehensive examinations, the origin of the patient’s metastatic tumor remains unidentified. Although some metastatic patterns may be used to speculate the primary site of the metastatic cancer, this strategy may not fully applicable to CUP due to its heterogeneity and unclear characterization. Therefore, we suggest that physicians should not speculate the primary site of CUP by the known metastatic patterns. In our case, a pelvic mass was initially detected by ultrasound. Although ultrasound is a common imaging technique that can show the location of tumors, it is not yet available for the specific diagnosis of pelvic tumors. In the case of this study, the patient was presumed to have rectal cancer with pelvic lymph node metastasis after preoperative multidisciplinary diagnosis. This is because metastases are concentrated in the lymph nodes and connective tissue around the pelvis. CT scan has the advantages of high resolution, clear image, clear anatomical relationship and assisting qualitative diagnosis. In this case, contrast-enhanced CT revealed a soft tissue mass in the left posterior part of the bladder, involving the left ureter and possibly invading the posterior wall of the bladder. The most common imaging tests are positron emission tomography CT (PET-CT), magnetic resonance imaging (MRI), and bone scan. PET-CT is helpful for the early diagnosis of pelvic tumors. It can stage the tumors, assess whether the tumor is recurrent. The most important thing is to help find the primary tumor and metastases. MRI is better than CT for finding pelvic organ tumors, especially for bladder, rectum, uterus and other parts of the examination. MRI also helps to determine the relationship of the tumor to other pelvic organs, which is important for the specific way the surgery is performed. At the same time, bone scanning can help us to detect early whether the pelvic tumor is the primary site or metastases from other sites. All aspects of the patient’s information should be collected as much as possible before surgery, and comprehensive examinations such as PET-CT, MRI, bone scan, and genetic testing should be performed to facilitate better understanding the characteristics of the tumors. Moreover, urinary-related tumor markers were basically normal. Since routine preoperative evaluation and further evaluation after operation failed to clarify the primary lesion, the malignancy was diagnosed as CUP after multidisciplinary comprehensive evaluation, including CT, abdominal ultrasound, prostate specific antigen value and urologist’s discussion. Since histological diagnosis is very important for the final decision on the treatment of CUP,^[10]^ the patient was also examined by immunohistochemistry (hematoxylin and eosin stain staining). However, the final pathological examinations suggested that it should be poorly differentiated pelvic squamous cell carcinoma. Nonetheless, we suggest that multiple possibilities should be considered when facing pelvic tumor patients. It is better to collect relevant information about the possible primary site of tumor and exclude common primary sites before operation. In addition, a definitive diagnosis should be made to facilitate treatment.

In addition to the difficulty in identifying the primary tumor origin of CUP, the treatment of CUP in clinical practice is also extremely challenging. Despite several phase 3 clinical trials for CUP treatment, there is currently no consensus on CUP treatment.^[11]^ In clinical practice, surgery is usually the first option for the treatment of CUP. However, if the prognosis of CUP is considered poor due to the lesion sites and pathological characteristics, surgery followed by chemotherapy is the main treatment. Because CUP cannot initially assess the primary site, there is no uniform chemotherapy and immunotherapy regimen for CUP patients. Only routine chemotherapy or immunotherapy can be given based on pathological results in related reports.^[12]^ For surgical methods, total resection was significantly associated with improved survival. For patients with squamous cell carcinoma and present lymph node metastasis, lymph node dissection is recommended. If the lesion is localized, preoperative chemotherapy can be considered, of which platinum-based chemotherapy is usually effective. A recent study reported that programmed cell death protein 1 based immunotherapy showed clinical benefits in solid tumor patients with unresectable or metastatic MSI-H/dMMR.^[13]^ However, a comprehensive survey of CUP revealed that the proportion of CUP cases harbored genetic alterations in MSI-H/dMMR is very low.^[14]^ Therefore, if programmed cell death protein 1 immunotherapy is used in CUP patients, the effect may not be ideal. In this study, when the patient with pelvic tumor was diagnosed as a CUP, there were already multiple pelvic lymph node metastases. After operation, the surgical margin basically reached the level of R0 resection, and the compression dilation of the left ureter and hydronephrosis of the patient were significantly relieved. However, the patient refused postoperative chemotherapy and immunotherapy. The patient died 3 months after surgery due to disease progression. According to the treatment experience of this CUP case, surgical resection of the focal tissue alone may not improve the prognosis of CUP. We recommend that patient with a CUP should continue to receive subsequent chemotherapy and immunotherapy after surgical resection to improve survival.

Due to the lack of pathological features, the primary origin of pelvic tumors depends on multiple imaging modalities and histological examinations. According to the pathological findings of the metastatic squamous cell carcinoma, its primary origin is mostly like to originate from lung or bladder. In this case, we did not first consider the bladder as the primary site because CT did not report a mass in the bladder. In addition, the patient finally chose to remove the tumor tissue through surgery. Based on these 2 points, cystoscopy or ureteroscopy was not performed. Despite the multiple diagnostic methods available today, pelvic tumors are still difficult to detect. Imaging alone cannot identify the primary tumor, so histological examination is necessary. For instance, in many clinical practices, the primary site of malignancy cannot be determined even with pathological examination of the surgically resected tissue. Therefore, for patients whose primary tumor site cannot be determined, we suggest that these patients should continue other treatment approaches after surgical resection, such as conventional chemotherapy and radiotherapy, as well as immunotherapy and targeted therapy that are currently considered to have low side effects and are well tolerated. We also need to obtain more clinical data from other CUP patients who received various treatments, so that we will have more experience to improve the diagnosis and treatment for patients with CUP.

## 4. Conclusion

The treatment mode of CUP is still in the process of empirical treatment, and no consensus has been reached. This case therefore suggests that pelvic tumors should be removed as soon as they are found. Even if it has progressed to the middle or late stages, surgical resection should be considered as early as possible before severe organ compression symptoms appear. Although, the patient with CUP in our hospital received surgical treatment actively, the disease continues to progress. In spite of the pain symptoms were relieved, the postoperative survival was too short, only 3 months. Surgical treatment alone cannot improve the poor prognosis of CUP. With the accumulation of various treatment experience, we should be able to share more diagnostic options and therapeutic treatment for such rare diseases.

## Acknowledgements

We thank the patient reported in this paper, and his family for the assistance. We also thank Dr Chien-Chang Huang for providing professional English language editing.

## Author contributions

**Conceptualization:** Wenjun Meng, Yuchen Gao, Lian Bai.

**Data curation:** Wenjun Meng, Yuchen Gao, Guowei Zhao, Qi Chen.

**Funding acquisition:** Rujun Zheng.

**Methodology:** Wenjun Meng, Yuchen Gao.

**Supervision:** Lian Bai, Rujun Zheng.

**Writing – original draft:** Wenjun Meng, Yuchen Gao, Lu Pan.

**Writing – review & editing:** Lian Bai, Rujun Zheng.
